# Physicochemical and microbiological characteristics of pork meat exposed to SoRegen^®^ Technology

**DOI:** 10.14202/vetworld.2025.484-490

**Published:** 2025-02-26

**Authors:** Listya Purnamasari, Joseph Flores dela Cruz, Chang Soo Kim, Seong Gu Hwang, Jun Koo Yi

**Affiliations:** 1 Department of Animal Husbandry, Faculty of Agriculture, University of Jember, Jember, 68121, Indonesia; 2 Department of Basic Veterinary Sciences, College of Veterinary Medicine, University of the Philippines Los Banos, Laguna, 4031, Philippines; 3 Research Council of SoRegen Convergence Science, Seoul, Republic of Korea; 4 School of Animal Life Convergence Science, Hankyong National University, Anseong, 17579, South Korea

**Keywords:** cholesterol, microbiological safety, physicochemical properties, pork meat, quantum entanglement, SoRegen^®^ Technology

## Abstract

**Background and Aim:**

Quantum entanglement has been explored as a novel approach in food technology to enhance the quality and nutritional properties of animal products. SoRegen^®^ Technology applies quantum entanglement signals to food products, aiming to induce physicochemical modifications. This study investigates the effects of SoRegen^®^ Technology on the physicochemical and microbiological characteristics of pork meat, focusing on meat quality attributes and cholesterol levels.

**Materials and Methods:**

Pork meat samples were exposed to a quantum entanglement signal from a SoRegen^®^ chip for 20 min in an electromagnetic field chamber. Physicochemical properties, including water-holding capacity (WHC), pH, drip loss, and cooking loss, were analyzed. Cholesterol levels, including total cholesterol and high-density lipoprotein (HDL) cholesterol, were measured using enzyme-linked immunosorbent assay kits. Microbiological analysis included total plate count (TPC), *Escherichia coli*, and *Listeria monocytogenes* enumeration. Data were statistically analyzed using an independent sample t-test with significance set at p < 0.05.

**Results:**

Pork meat exposed to SoRegen^®^ Technology exhibited significant improvements in physicochemical properties. WHC increased, indicating better moisture retention, while drip loss and cooking loss were reduced, suggesting improved meat texture and quality. Minor but statistically significant fluctuations in pH were observed at specific time points. Cholesterol analysis revealed a significant 35% reduction in total cholesterol levels, alongside a significant increase in HDL cholesterol levels, indicating potential health benefits. Microbiological analysis indicated no significant differences between exposed and unexposed pork samples in terms of *E. coli*, *L. monocytogenes*, and TPC, suggesting that the technology does not directly affect microbial contamination.

**Conclusion:**

The application of SoRegen^®^ Technology significantly improved the physicochemical and nutritional attributes of pork meat, particularly by enhancing WHC and reducing cholesterol levels. However, no significant changes were observed in microbiological characteristics. These findings highlight the potential of quantum entanglement technology in food science, though further research is required to elucidate the mechanisms underlying these effects and explore commercial applications.

## INTRODUCTION

The global pig industry is responsible for pork production, accounting for approximately 33% of total meat consumption in 2021 [1]. The wide variety of pork products that create specific quality expectations for the “raw” materials used for processing, combined with the growing expectations of consumers for more sustainable pig production systems, makes pork quality a complex and multidimensional issue. During storage, fresh food products are affected by microbial spoilage, enzyme activity, and physical and chemical changes due to intrinsic and extrinsic factors, such as food composition, temperature, and humidity [2]. In particular, the major influence of dietary fatty acid composition, particularly cholesterol, on the human consumption of meat has been proposed as a dietary guideline for decades.

Cholesterol is highly hydrophobic and exists in the form of lipoproteins, which must be transported in association with proteins. There are two main forms of cholesterol, depending on the density of the proteins. Low-density lipoprotein (LDL) is known as bad cholesterol, and high-density lipoprotein (HDL) is known as good cholesterol [3]. LDL can cause fatty deposits, called plaques, on the walls of blood vessels, narrowing the arteries, straining blood flow, and increasing the risk of cardiovascular disease and stroke [4]. HDL can reduce the risk of heart attack and stroke due to its ability to reduce plaques in blood vessels and absorb cholesterol, bringing it to the liver for oxidation or metabolism by bile [5]. To reduce excessive cholesterol levels, consumers attempt to reduce their intake of trans fats, particularly animal products. Therefore, it would be interesting to identify preservation technologies for meat quality and the nutritional aspects that are key to food quality.

Since the Nobel Prize in Physics was awarded in 2022 for a new study on quantum entanglement, the behavior of atomic nuclei and electrons has become very interesting. The physics of quantum entanglement reveals that information is constantly entangled in spooky actions and is transmitted infinitely faster than light [6]. Research Council of SoRegen Convergence Science, Republic of Korea, introduced a new technology called SoRegen® Technology. SoRegen® Technology proposed that exposure to a quantum entanglement signal program improves the function and characteristics of materials. This technology related to magnetic fields is not clearly presented in terms of introducing a quantum effect similar to that of SoRegen**^®^** wave information and applying it to quantum biological effects of exposure to wave information. By applying this quantum entanglement theory, we propose the possibility of changing materials by exposing radio electromagnetic waves by introducing SoRegen^®^ Technology [7]. A previous study by Purnamasari *et al*. [8] showed that SoRegen^®^ Technology water had anti-inflammatory activities with potential clinical immunomodulatory effects. Quantum superposition refers to the remarkable ability of small particles to exist simultaneously in multiple states. This concept is related to various biological and physical processes and intriguing phenomena. The application of changing materials in tissues by exposing pork meat to radio-electromagnetic radiation could impact the nutritional quality of meat; therefore, further investigation is needed. To investigate changes in biological effects, meat samples were exposed to a specific quantum entanglement signal from the SoRegen^®^ chip.

This study aimed to assess the possibility of producing new functional foods by introducing SoRegen^®^ Technology and measuring and analyzing changes in the properties and contents of healthy functional ingredients in animal products.

## MATERIALS AND METHODS

### Ethical approval

This study analyzed the physicochemical and microbiological characteristics of pork exposed to SoRegen® Technology. The pork samples were imported from Teyo Food Llc, Mexico, by GY Food Co Ltd, South Korea, with registration number 89-2CG2023600567675 and quarantine inspection number 13-ISMD2309200301.

### Study period and location

The study was conducted from January to December 2023 at the Applied Biochemistry Laboratory, Hankyong National University, South Korea. Exposing meat samples to electromagnetic field signals generated by a SoRegen® chip was conducted at the Research Council of SoRegen Convergence Science, Seoul, Republic of Korea.

### SoRegen^®^ Technology

**Figure 1:**
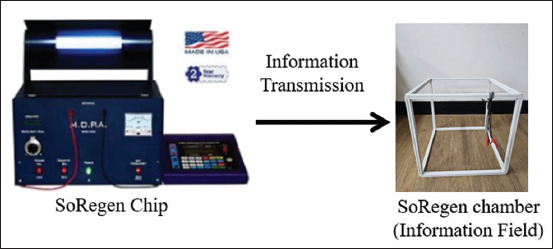
The information transmission procedure.

Pork belly samples were exposed to a specific quantum entanglement signal from a SoRegen**^®^** chip for 20 min in an electromagnetic field chamber (Figure 1). The molecular structure changes through electron rearrangement by receiving the information wave field signal inside the chamber and resonating the information with the meat, causing quantum entanglement phenomena.

### Total cholesterol

The total cholesterol level in pork meat was analyzed using commercial enzyme-linked immunosorbent assay (ELISA) kits (My BioSource, MBS 168179, USA). HDL levels were also analyzed using commercial ELISA kits (Cell Biolabs, Inc. STA-391, USA). All analyses were performed according to the manufacturer’s instructions, and absorbance was measured using a spectrophotometer (Microplate Reader, Tecan Infinite F50 Instrument, Switzerland).

### Physicochemical characteristics of meat

#### Drip loss

The natural drip was measured after storage for 1, 3, 7, or 14 days in a refrigerator at 4 ± 0.5°C. The initial and final weights were obtained, and the drip loss was calculated.

Drip loss was calculated as a percentage of weight loss as follows:


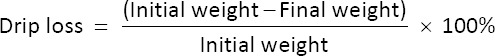


### Meat pH

The pH of the meat was measured with a pH meter after calibration by dipping its head into a neutral buffer solution (pH = 7.00) and then into a calibration solution (pH = 4.00). For this purpose, 10 g of meat was weighed and 90 mL of distilled water was added, after which the meat was chopped using a meat chopper. The pH was measured 3 times and the mean value was determined.

### Water-holding capacity (WHC)

The WHC of the samples was measured using centrifugal force. The Whatman filter papers number 3 (WHA1003110, Merck, Germany) were cut into four pieces. Then, 1.5 g of meat sample was weighed (W0) and then covered with one piece (i.e., a quarter) of the filter paper, placed in a 50 mL tube, centrifuged at 3000 rpm, and then weighed again (W1). Finally, the WHC was computed using the following formula:

WHC (%) = (100 – [(W0-W1)/W0] × 100)

### Cooking loss

Cooking loss was evaluated by weighing 20 g of meat sample (W0) into a 50 mL tube followed by centrifugation at 3000 rpm for 10 s and heating in a water bath at 85°C for 45 min. The cooked samples were then cooled and weighed (W1). Finally, the cooking loss was computed using the following formula:

Cooking loss (%) = ([W0-W1]/W0 × 100)

### Microbiological analyses

#### Total plate count (TPC)

Briefly, 25 g of meat sample was placed in a sterile filter bag, and 225 mL of normal saline was added. This was then homogenized with a stomacher for 1 min, followed by a 3-fold serial dilution. The dilutions (1 mL) were spread on agar plates (Plate Count Agar, MB-P1040, Korea), maintained at 37°C for 48 ± 2 h, and then the TPC was recorded.

#### Escherichia coli

Five-fold serial dilutions of stomached samples were plated (1 mL) on agar plates (MacConkey Sorbitol Agar, MB-M1108, Korea). All samples were incubated at 37°C for 24 h before enumeration.

#### Listeria monocytogenes

Three-fold serial dilutions of stomached samples were plated (1 mL) on agar plates (Oxford Agar, MB-O1310, Korea). All samples were incubated at 37°C for 24 h before enumeration.

### Statistical analysis

All data were analyzed using IBM Statistical Package for the Social Sciences Statistics 24 (IBM Corp., Armonk, NY, USA). The results are presented as mean ± standard deviation. The normality of the data distribution was assessed using the Shapiro–Wilk test, and the homogeneity of variances was verified using Levene’s test. An independent sample t-test was performed to compare the physicochemical, cholesterol, and microbiological characteristics of pork meat between the SoRegen^®^-treated and untreated groups. p < 0.05 was considered statistically significant.

For cholesterol and HDL levels, the differences between treated and untreated samples were analyzed using an independent sample t-test. Physicochemical properties such as WHC, drip loss, cooking loss, and pH changes over different storage periods were also compared using an independent sample t-test. Microbiological analyses, including TPC, *E. coli*, and *L. monocytogenes*, were evaluated similarly.

All statistical tests were conducted at a 95% confidence interval, and the results were interpreted accordingly. Data were visualized using descriptive statistics and graphical representation where necessary to highlight significant trends.

## RESULTS

After slaughter, biochemical changes that cause the conversion of muscle to meat determine the final meat quality. Further processing has become a matter of concern for the nutritional quality of pork meat. Table 1 lists the physicochemical characteristics of pork meat after exposure to a specific quantum entanglement signal from a SoRegen**^®^** chip for 20 min in an electromagnetic field chamber. The results showed that pork meat exposed to a specific quantum entanglement signal from the SoRegen**^®^** chip significantly improved the quality of the meat compared to unexposed meat.

**Table 1:** Physicochemical characteristics of pork meat.

Item	Normal pork	Treatment pork	p-value
pH			
1*	6.12 ± 0.04	6.20 ± 0.11	0.070
3*	6.26 ± 0.07	6.13 ± 0.10	0.008
7	6.10 ± 0.04	6.08 ± 0.05	0.447
14*	6.47 ± 0.07	6.53 ± 0.02	0.036
Drip loss (%)			
1*	6.45 ± 0.10	5.86 ± 0.18	0.000
3*	6.92 ± 0.21	6.34 ± 0.35	0.012
7*	7.21 ± 0.17	6.83 ± 0.15	0.006
14*	7.59 ± 0.21	7.21 ± 0.14	0.009
Cooking loss (%)			
1	24.32 ± 0.94	22.24 ± 1.10	0.068
3	24.39 ± 0.66	22.75 ± 0.89	0.063
7	24.47 ± 0.50	22.89 ± 0.91	0.058
14	25.93 ± 0.99	24.05 ± 0.70	0.054
Water-holding capacity (%)			
1*	77.98 ± 0.60	80.16 ± 0.78	0.018
3	75.85 ± 1.20	78.44 ± 1.26	0.062
7*	73.54 ± 0.40	75.99 ± 1.06	0.020
14*	71. 38 ± 1.03	73.37 ± 0.37	0.035

Means in the same row with (*) indicate significant differences (p < 0.05). Data are presented as mean ± standard deviation. Means were calculated using three replications per treatment (n = 9).

Tables 2 and 3 present the total cholesterol and HDL levels in pork meat exposed to a quantum entanglement signal from the SoRegen**^®^** chip. The total cholesterol level in exposed pork meat was significantly lower than that in unexposed pork meat (p < 0.05). A reduction of approximately 35% was observed after treatment with SoRegen**^®^**. In contrast, the HDL cholesterol content increased significantly after exposure to a quantum entanglement signal from the SoRegen**^®^** chip (p < 0.05).

**Table 2:** Total cholesterol level of pork meat.

Total cholesterol	Normal	SoRegen®	p-value
(μM)*	647.07 ± 167.61	359.73 ± 180.53	0.031
(mg/dL)*	24.98 ± 6.47	13.89 ± 6.97	0.031

Means in the same row with (*) indicate significant differences (p < 0.05). Data are presented as mean ± standard deviation. Means were calculated using three replications per treatment (n = 9)

**Table 3:** High-density lipoprotein cholesterol levels in pork meat.

Total cholesterol	Normal	SoRegen®	p-value
(mmol/L)*	0.10 ± 0.06	0.27 ± 0.08	0.043
(mg/dL)*	1.89 ± 1.05	4.84 ± 1.47	0.047

Means in the same row with (*) indicate significant differences (p < 0.05). Data are presented as mean ± standard deviation. Means were calculated using three replications per treatment (n = 9).

Table 4 presents the total microbiology of pork meat after exposure to a quantum entanglement signal from the SoRegen**^®^** chip for 20 min in an electromagnetic field chamber. There were no significant differences in the pork meat between the exposure and unexposed treatments.

**Table 4:** Total microbiology of pork meat.

Item	Normal pork	Treatment pork	p-value
*Escherichia coli* (10^5^ CFU/g)			
1	1.23 ± 0.48	1.12 ± 0.58	0.820
3	6.60 ± 4.61	5.35 ± 0.78	0.667
7	11.66 ± 10.57	9.69 ± 4.80	0.784
14	113.4 ± 17.0	93.68 ± 15.61	0.213
*Listeria monocytogenes* (10^5^ CFU/g)			
1	0.01 ± 0.001	0.01 ± 0.002	0.967
3	0.03 ± 0.005	0.02 ± 0.007	0.992
7	0.10 ± 0.04	0.08 ± 0.02	0.533
14	0.15 ± 0.02	0.13 ± 0.02	0.377
TPC (10^5^ CFU/g)			
1	0.33 ± 0.06	0.35 ± 0.09	0.971
3	0.10 ± 0.06	0.08 ± 0.05	0.623
7	2.42 ± 0.02	1.58 ± 1.28	0.444
14	>3.5	>3.5	

Means in the same row with (*) indicate significant differences (p < 0.05). Data are presented as mean ± standard deviation. Means were calculated using three replications per treatment (n = 9). CFU=Colony-forming unit, TPC=Total plate count

In this study, the physicochemical characteristics of pork meat were improved after exposure to a specific quantum entanglement signal from the SoRegen**^®^** chip for 20 min in an electromagnetic field chamber compared with the unexposed treatment. The total cholesterol content of meat was reduced and HDL cholesterol content increased after exposure to the SoRegen**^®^** Technology.

## DISCUSSION

The essential physical parameters for assessing the quality of pork meat are its pH, drip loss, cooking loss, and WHC [9]. Because refrigeration conditions offer a narrow shelf life, freezing is an alternative preservation method that can prolong the shelf life of products [10]. Ice crystal formation can deform the cellular structure, affecting the texture and color as well as causing internal damage to the structure and physicochemical characteristics of food products including meat. Both particle and wave electromagnetic fields affect the activity of electrons and photons, causing changes in their physicochemical properties. These phenomena are caused by quantum mechanical applications to target organs and suppress ice crystal growth in pork meat. New technologies such as high-pressure and ultrasound can improve hygienic quality and meat tenderness [11]. Water tightly binds to muscle proteins, resulting in swelling. It also occupies the spaces between myofibrils and gives the meat a firmer structure. The rate and extent of chemical and physical changes that occur in the muscle also determine the tenderness of the meat [12].

The ice crystal formation rate depends on the freezing rate and the air flow rate or air circulation around the sample [13]. Numerous methods have been developed to suppress ice crystal growth in pork meat, including rapid freezing [14], ultrasound-assisted immersion freezing [15], oscillating magnetic field freezing [16], static electric field freezing [17], and magnetic resonance freezing [18]. Some of these technologies are used in industry, whereas others are limited to laboratory results.

Cholesterol is a structural component of cell membranes and a building block for synthesizing various steroid hormones [19], and it is utilized for the absorption of fat-soluble vitamins (A, D, E, and K) and bile acids [20]. Cholesterol plays an important role in the regulation of cellular functions. It can be found in the blood and is necessary for healthy cells; however, high levels of cholesterol can be deposited in blood vessels, impairing the circulation of blood through the arteries, increasing blood pressure, and causing the rupture of capillaries that increase the risk of stroke or cardiovascular disease [21].

In various fields, such as radiobiology, toxicology, and environmental health, exposure to wave energy acts as a stressor in biological systems, and the harmful effects of ionizing radiation accumulate in organisms and cause widespread damage [22]. Although not directly targeted, these phenomena have been reported to cause changes in signal transduction systems and gene instability at near or far distances, either directly or indirectly [23]. Quantum superposition refers to the remarkable ability of small particles to exist simultaneously in multiple states. This concept is related to various biological and physical processes and intriguing phenomena. In addition to light from the solar system, organisms live in the resonance of many invisible waves of energy, including radio waves, electromagnetic waves, infrared waves, ultraviolet waves, and static electricity.

Microbial contamination of pork can occur at any stage, from pig slaughter to pork distribution in retail outlets and during handling in households [24]. Pathogenic microorganisms, such as *E. coli* and *L. monocytogenes*, cannot be detected with the naked eye [25] and are considered the most dangerous food pathogens in terms of the number of deaths they cause [26]. The food safety conditions of pork meat contribute significantly to microbial contamination. During production, pork meat should be kept at cool temperatures to slow bacterial growth and limit further contamination [27]. The European Union recommends that the levels of contamination by total bacteria and total coliforms should not exceed 5.0 and 2.5 log colony-forming unit/g, respectively [28], which are classified as low risk in terms of the transmission of pathogenic bacteria to consumers. Temperature is the major factor affecting microbial growth, and its control during storage can reduce meat spoilage. The effects of temperature on microbial growth have been extensively studied [29]. The application of SoRegen^®^ Technology can improve consumer health. However, no significant difference in pork meat was observed between the exposure and unexposed treatments. This research proposes a new turning point in the food industry in which quantum biological effects are applied.

There are many different forms of wave energy on earth, and the biological responses observed in cells or tissues exposed to these energies without specific targeting are as pronounced as those observed in stressors such as ionization from targeted exposure, radiation, or chemicals. Recently, it has been reported that the amount of intracellular reactive oxygen species (ROS) produced depends on the frequency or amplitude of exposure to radio electromagnetic waves. ROS production is mediated through intracellular receptors and leads to biological changes, such as intracellular oxidative stress, simulation, or inhibition of cell signaling and response systems [30]. These findings suggest that in complex biological systems, physiological processes occurring within biomolecules, such as proteins and genetic material, are closely related to quantum effects, affecting biological functions, including enzyme activity regulation, magnetic field sensing, cellular metabolism, and electron transfer.

## CONCLUSION

This study investigated the effects of SoRegen^®^ Technology, which applies quantum entanglement signals, on the physicochemical and microbiological characteristics of pork meat. The findings demonstrated significant improvements in meat quality attributes, particularly in WHC, drip loss, and cooking loss. WHC increased significantly (p < 0.05), contributing to improved meat texture and juiciness, while drip loss and cooking loss were notably reduced, indicating better moisture retention and reduced structural degradation. Minor but statistically significant fluctuations in pH were observed at certain time points.

In terms of cholesterol content, the total cholesterol level in pork meat exposed to SoRegen^®^ Technology was significantly reduced by approximately 35% (p < 0.05), while HDL cholesterol levels increased significantly (P < 0.05). These results suggest that quantum entanglement exposure may have beneficial effects on the nutritional profile of meat, particularly in reducing cholesterol-related health risks. However, microbiological analysis indicated no significant differences between treated and untreated meat samples for *E. coli*, *L monocytogenes*, and TPC, suggesting that SoRegen^®^ Technology does not directly affect microbial contamination.

One of the strengths of this study is the novel application of quantum entanglement in food science, offering a non-invasive method for enhancing meat quality and nutritional properties without the use of chemicals or additives. The study provides valuable insights into potential advancements in meat processing and preservation using wave-based information technology. However, a key limitation is the lack of mechanistic understanding of how quantum entanglement affects physicochemical properties at the molecular level. In addition, the study was limited to short-term effects, and long-term stability of the observed changes was not assessed.

Future research should focus on elucidating the underlying biochemical mechanisms responsible for the observed improvements in meat quality and cholesterol content. Further investigations are needed to assess the long-term stability, safety, and commercial viability of SoRegen^®^ Technology in meat processing. Expanding the study to different types of meat and food products could help validate the broader applicability of this technology. In addition, integrating advanced imaging and molecular techniques could provide deeper insights into the structural and compositional changes induced by quantum entanglement exposure.

While the findings indicate promising potential for quantum entanglement technology in food science, further rigorous studies are required to establish its efficacy, safety, and applicability in commercial food processing.

## AUTHORS’ CONTRIBUTIONS

SGH, LP, and CSK: Conceived and designed the study. LP and JFC: Collected and analyzed the data and performed the experiments. LP and JKY: Analyzed the data and drafted the manuscript. LP: Statistical analysis. LP, JFC, and SGH: Interpreted the data and revised and finalized the manuscript. All authors have read and approved the final manuscript.
